# A Distributed and Context-Aware Task Assignment Mechanism for Collaborative Mobile Edge Computing

**DOI:** 10.3390/s18082423

**Published:** 2018-07-25

**Authors:** Bo Gu, Yapeng Chen, Haijun Liao, Zhenyu Zhou, Di Zhang

**Affiliations:** 1 School of Electrical and Electronic Engineering, North China Electric Power University, Beijing 102206, China; bo.gu@cc.kogakuin.ac.jp (B.G.); 15601258900@163.com (Y.C.); NellyLiao31@163.com (H.L.); 2 Department of Information and Communications Engineering, Kogakuin University, Tokyo 192-0015, Japan; 3 School of Information Engineering, Zhengzhou University, Zhengzhou 450052, China

**Keywords:** edge computing, fog computing, cloud computing, energy efficient, task assignment, MEC, vehicle, matching, stability, intelligent computation, QoS, utility, preference.

## Abstract

Mobile edge computing (MEC) is an emerging technology that leverages computing, storage, and network resources deployed at the proximity of users to offload their delay-sensitive tasks. Various existing facilities including mobile devices with idle resources, vehicles, and MEC servers deployed at base stations or road side units, could act as edges in the network. Since task offloading incurs extra transmission energy consumption and transmission latency, two key questions to be addressed in such an environment are (i) should the workload be offloaded to the edge or computed in terminals? (ii) Which edge, among the available ones, should the task be offloaded to? In this paper, we formulate the task assignment problem as a one-to-many matching game which is a powerful tool for studying the formation of a mutual beneficial relationship between two sets of agents. The main goal of our task assignment mechanism design is to reduce overall energy consumption, while satisfying task owners’ heterogeneous delay requirements and supporting good scalability. An intensive simulation is conducted to evaluate the efficiency of our proposed mechanism.

## 1. Introduction

The explosive growth of information and communication technologies has spurred an array of computation-intensive and delay-sensitive applications, such as augmented reality (AR) and virtual reality (VR) automotive applications, and allowed new functionalities, like self-driving vehicles, with the final aim of enriching our lives. However, despite the great advances that might be brought by the promising applications, several challenging issues need to be addressed in advance.

Computation-intensive applications generally require a high computing capacity for data processing which cannot be easily offered by mobile terminals (e.g., smart phones and wearable devices) due to their limited resources. Cloud computing [1] enables on-demand network access to a shared pool of configurable computing resources which can largely augment on-device computing capacity. By 2021, it is predicted that 94% of the total workload and compute instances will be processed in the cloud, and the annual IP traffic originated or terminated at data centers will reach 20.6 Zettabytes (ZB) by the end of 2021 [2]. However, the transmission of such huge volume of data to cloud data centers will not only pose a heavy burden on the capacity-constrained backhaul and backbone networks, but will also result in unpredictable transmission latency and a degraded Quality of Service (QoS) to end users. To this end, mobile edge computing (MEC) has been proposed as a promising paradigm to address the latency issue by switching from the conventional centralized cloud computing architecture to a distributed one [3]. Various existing facilities, such as mobile devices with idle resources, vehicles, and MEC servers deployed at base stations or road side units, could act as distributed edges [4]. Offloading computation tasks to such edges that are physically close to the data sources could significantly reduce the transmission latency and hence, improve the QoS perceived by end users.

However, task offloading in MEC architecture is highly challenging. This is owing to the mobility of end devices and vehicles, the inherent environmental dynamics associated with the communication medium, such as intermittent wireless connectivity, and the heterogeneous computational capabilities of edges and the heterogeneous QoS requirements of computation tasks. Moreover, the reality that both parties (i.e., tasks and edges) usually do not have complete information about each other complicates the problem even further.

Theoretically, optimal task offloading can be achieved when all task owners and edges share their information (e.g., mobility patterns, delay constraints, measured channel gains, computational capabilities, etc.) to a centralized authority. After gathering all the information, the centralized authority can then determine (i) whether to offload the task to the edge or compute it in terminals, and (ii) which edge, among the available ones, should the task be offloaded to. Obviously, such a centralized scheme for task offloading requires excessive information exchange which is therefore impractical in real MEC systems.

Energy efficiency is another mainstream concern for the design of task offloading mechanism in MEC architectures. By 2020, more than 50 billion mobile devices are expected to be connected and exchanging information with each other; the subsequent energy consumption and CO${}_{2}$ emissions will account for about 4% of global emissions. In addition to the environmental concerns, mobile devices are usually battery operated, which makes saving energy consumption a non-trivial challenge in MEC.

In this paper, we formulate the task assignment in MEC to a one-to-many matching problem by taking into account the devices’ and MEC servers’ computation capabilities, wireless channel conditions, and delay constraints. The main goal of our task assignment mechanism design is to reduce the overall energy consumption, while satisfying task owners’ heterogeneous delay requirements and supporting good scalability.

The contributions of this paper are summarized as follows:

- First, we study the problem of task assignment in MEC. The proposed task assignment mechanism is context-aware, and the task assignment is optimality-driven and can be performed in a distributed manner.
- Second, we theoretically prove that the proposed task assignment mechanism results in stable matching between the tasks and edges; namely, there are no task-edge pairs who both prefer each other over their current match.
- Third, our proposed task assignment mechanism can significantly reduce the overall energy consumption and provides a good compromise between computational complexity and energy consumption optimization. Simulation results confirm that, by dispatching tasks to edges carefully, we can achieve better system performance.

The rest of this paper is organized as follows. In Section 2, we give a brief literature review. In Section 3, we present our problem formulation. In Section 4, we propose a matching-theory based solution and analyze its stability. In Section 5, we conduct intensive simulations to confirm the efficiency of our proposed mechanism. Finally, in Section 6, we conclude our paper and identify several open issues that will be addressed in our future work.

## 2. Related Work

As one of the most promising technologies for improving the computing experience of end users, MEC has attracted considerable attention from both industry and academia since its inception. From the industry side, in 2013, IBM and Nokia Siemens Networks announced the very first MEC platform named Radio Applications Cloud Server (RACS) [5], which can run applications directly within a mobile base station. But the real momentum was achieved in 2014, when an Industry Specification Group (ISG) focusing on MEC was started in ETSI to create consensus within the ecosystem on a first set of MEC specifications, and since then, several companies have proposed semi-proprietary solutions. Arm’s Mbed Edge, offers a transparent distributed application execution environment to enable processing of rules and data on gateways, thus enabling a wide range of IoT devices to be monitored and controlled remotely via gateways. Microsoft’s Azure IoT Edge further integrates machine learning and artificial intelligence (AI) at gateways which allows the deployment of advanced analytics, event processing, and image recognition services without accessing the cloud.

In recent years, many research efforts have focused on MEC as well. In reference [6], Luan et al. pointed out that providing virtualized resources and localized services to the edge of mobile networks can better cater to the versatility of requirements for end users. In reference [7], Shi et al. presented several MEC-based use cases, including video analytics, smart home, smart city, and collaborative edge. In reference [8], Tran et al. demonstrated the consequent benefits of integrating MEC into the 5G ecosystem and then discussed the key technical challenges in terms of resource management and mobility support, etc. In reference [9], Gu et al. argued that cloud resources can be utilized to process medical sensor data while the unstable and high-latency links between the cloud and medical smart devices impede the development of medical cyber-physical systems (MCPSs). Then, they proposed a MEC-based solution to combat those issues. In reference [10], Truong et al. extended MEC into vehicular ad hoc networks (VANETs) and proposed an advanced framework for VANETs that integrates the flexibility, scalability, and programmability of Software Defined Networking (SDN) and the low latency, mobility support, and location awareness enabled by MEC. In reference [11], Hou et al. proposed the concept of utilizing moving and parked vehicles as the infrastructures for communication and computation (VFC), and showed that the computational performance could be largely improved by exploiting excessive computing resources of individual vehicles. In reference [12], Zhou et al. proposed a dependable content distribution framework that combines big data-based vehicle trajectory prediction with coalition game-based resource allocation in cooperative vehicular networks. In reference [13], Baccarelli et al. introduced the concept of Fog of Everything (FoE), which exploits the advantages of both edge computing and the Internet of Everything (IoE) and provided several application use-cases ranging from smart city, Industry 4.0 to big data streaming. In reference [14], Zhou et al. proposed a price-based bandwidth slicing framework which could be applied in MEC to achieve efficient resource usage without requiring changes to existing protocols. In reference [15], He et al. proposed a deep learning-based offloading strategy to optimize the performance of IoT systems with edge computing.

Another thread of research has been focused on reducing the energy consumption of MEC. In reference [16], Zhang et al. proposed an energy-efficient computation offloading mechanism, which jointly optimizes task offloading and radio resource allocation so as to achieve minimum energy consumption under delay constraints. In reference [17], Deng et al. investigated the tradeoff between power consumption and transmission delay in the fog-cloud computing system. They formulated a workload allocation problem by taking into account the power consumption and service delay constraints and proposed an approximate approach-based optimal workload allocation policy. In reference [18], Liu et al. presented a Markov decision process approach for optimally scheduling stochastic tasks to achieve the objective of power-limited delay minimization. In reference [19], Zhou et al. proposed an energy-efficient resource allocation algorithm by jointly considering channel selection and power allocation in cloud radio access networks. In reference [20], Zeng et al. formulated task scheduling and task image placement in a fog computing supported software-defined embedded system (FC-SDES) to a mixed-integer nonlinear programming problem, and proposed a computation-efficient algorithm to find the optimal solution. In reference [21], Park et al. proposed an energy-efficient user association and power control policy. In reference [22], Zhou et al. proposed an energy-efficient matching mechanism for resource allocation in device-to-device (D2D) enabled cellular networks, which employs a game theoretical approach to formulate the interaction among end users, and adopted the Gale–Shapley algorithm to achieve stable D2D matching. In reference [23], Xu et al. proposed a novel game theoretical approach to encourage edge nodes to cooperatively provide caching services and reduce energy consumption. In reference [24], Tao et al. formulated an energy minimization problem for MEC systems and used convex optimization techniques to solve it. In reference [25], Sun et al. proposed a novel algorithm based on Lyapunov optimization and multi-armed bandit theories to achieve delay minimization under an energy consumption constraint. In reference [26] , Xu et al. proposed an in-memory storage and processing method to reduce energy consumption in a three-tier heterogeneous network architecture. In reference [27], Liu et al. proposed an energy-efficient duty cycle control scheme which intelligently adjusts the duty cycle of each node according to its energy consumption as well as the energy consumption of its neighbouring nodes. In reference [28], Huang et al. proposed a service routing-based caching scheme to balance the energy consumption among network devices and hence, increase the network lifetime.

Our work is different from the mentioned approaches, as we concentrate on task assignment in MEC systems and propose a distributed and context-aware task offloading mechanism to reduce the overall energy consumption while satisfying the delay constraints of tasks. In particular, all of the considerable operations are made based on end users’ local information which saves a significant amount of signaling overhead and hence, improves system scalability.

## 3. Problem Formulation

In this paper, mobile devices are classified into two categories: mobile devices with excessive computational capability, and those with limited computational capability. Note that all mobile devices may generate tasks but only those with excessive computational capability and MEC servers can accept offloaded tasks. For the sake of readability, mobile devices with excessive computational capability and MEC servers are collectively termed edge nodes (ENs).

**Figure 1:**
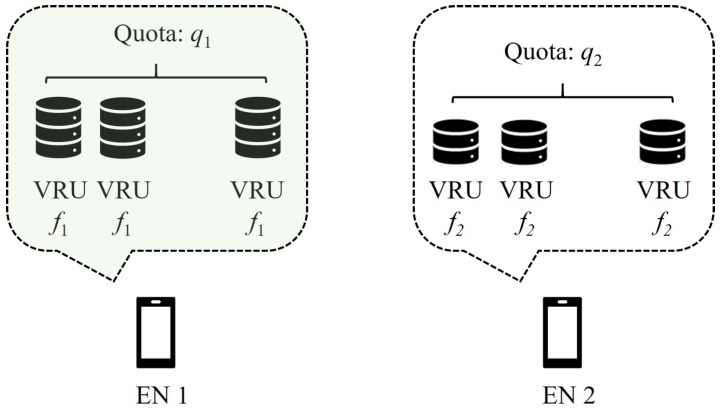
Heterogenous computational capacities.

Time is divided into multiple time slots. We assume that most tasks can be completed within one slot, while large-size tasks are divided into several sub-tasks so that they can also be completed in one slot [29]. The terminology “task“will be used to refer to both the task which is going to be computed as a whole in one slot and the divided sub-task in the rest of this paper. Furthermore, we assume that the number of tasks is *M* and the set of tasks is denoted by $\tau ={\left\{\tau_{i}\right\}}_{i=1}^{M}$. On the other hand, the number of ENs is *N* and the set of ENs is represented by $\epsilon ={\left\{\epsilon_{j}\right\}}_{j=1}^{N}$. ENs with high computational capability can divide their resources into several virtual resource units (VRUs) equally such that tasks can be computed in a parallel manner. The number of VRUs at EN *j* is referred to as the quota of EN *j*, which is denoted by $q_{j}$. The computational capability of VRUs at different ENs is assumed to be heterogeneous, as shown in Figure 1. We use the CPU frequency (in Hz) to describe the computational capability of each EN. Specifically, the CPU frequency of each VRU at EN *j* is denoted by $f_{j}$.

### 3.1. One-to-Many Matching

Matching theory is a useful tool for studyinf the formation of a mutual beneficial relationship between two sets of agents. In a matching game, each agent has its own preference and ranks the agents in the opposite set by using a utility function depending on some measurable parameters [30]. For example, in the classical college admission problem [31], students rank colleges according to their academic reputation, while the colleges rank the students according to their entrance examination scores, etc. Note that matching decisions are made by the agents themselves interactively based on their locally collected information. Therefore, matching theory-based protocols generally do not require any centralized coordinator and can support good scalability. Due to this advantage, matching theory is widely used in many fields of engineering. For instance, matching theory has been successfully used as a framework for studying user association problems in small cell wireless networks [32] and spectrum resource allocation problems in 5G networks [33,34].

**Figure 2:**
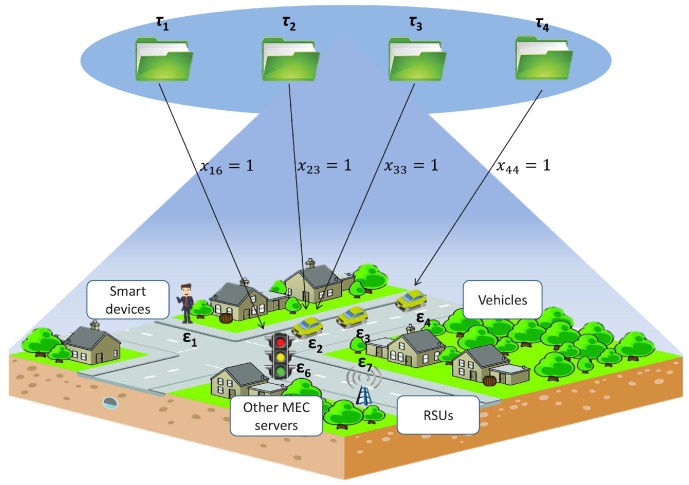
One-to-many matching.

To achieve a practical and distributed solution, we realize that the task assignment problem in MEC architectures can also be formulated as a matching game. As shown in Figure 2, computation tasks and ENs are considered to be two disjointed sets of agents to be matched together. In particular, one task can be assigned to, at most, one EN, while one EN can accept multiple tasks. We define the one-to-many matching function Φ as follows:

(1) $$\begin{array}{cc} & \Phi :\{\tau \}↔\{\epsilon \}\\ s.t.\; & (1)\;\Phi \left(\tau_{i}\right)\in \epsilon \;\mathrm{and}\;\left|\Phi \left(\tau_{i}\right)\right|\in \left\{0,1\right\}\\ & (2)\;\Phi \left(\epsilon_{j}\right)\subset \tau \;\mathrm{and}\;\left|\Phi \left(\epsilon_{j}\right)\right|\leq q_{j}\\ & (3)\;\Phi \left(\tau_{i}\right)=\epsilon_{j}\Leftrightarrow \Phi \left(\epsilon_{j}\right)=\tau_{i}. \end{array}$$

Condition (1) implies that each task could be assigned to, at most, one EN for execution; Condition (2) implies that each EN could accept at most its quota of tasks; and Condition (3) implies that if $\tau_{i}$ is matched to $\epsilon_{j}$, then $\epsilon_{j}$ is also matched to $\tau_{i}$.

We use a binary $x_{ij}$ to indicate whether task *i* is matched to EN *j* or not.

(2) $$x_{ij}=\left\{\begin{array}{cc} 1 & \mathrm{if}\;\Phi \left(\tau_{i}\right)=\epsilon_{j}\\ 0 & \mathrm{otherwise} \end{array}\right.,$$

where $x_{ij}=1$ implies that task *i* is matched to EN *j*, while $x_{ij}=0$ implies that task *i* is not matched to EN *j*.

### 3.2. Delay Constraint

First, we consider the delay constraint, which is a major concern that needs to be addressed in task assignment. In practice, different task owners have different time sensitivities. The metric of delay tolerance is a measure of a task owner’s time sensitivity, and it is defined as the time from when a computation request is made until the task is completed. The delay tolerance of each task, *i*, is denoted by $L_{i}^{\mathrm{tol}}$.

Since task offloading incurs extra transmission energy consumption and transmission latency, each task owner has to carefully decide whether to compute their task locally or offload it to neighboring ENs. In case of task offloading, the overall latency generally consists of three components: (i) transmission delay; (ii) queuing delay; and (iii) computation delay. First, the transmission delay refers to the time taken to transfer the input data or application context to a neighboring EN through a wireless connection. Second, the queuing delay is the time that a task waits in a queue until it can be executed. In this article, we assume that each task exclusively utilizes all resources of an EN or VRU for task execution. Therefore, queuing delay can be omitted. Finally, the computation delay is defined as the time taken to execute the task, which highly depends on the EN’s computation capability. In cases involving local computation, the transmission delay is 0.

Formally, the overall latency when task *i* is matched to EN *j* is denoted by $L_{ij}$ as follows:

(3) $$L_{ij}=L_{ij}^{com}+L_{ij}^{tra},$$

where $L_{ij}^{com}$, and $L_{ij}^{tra}$ represent the computational delay and transmission delay, respectively.

On one hand, the computational delay is given as follows:

(4) $$L_{ij}^{com}=\frac{C_{i}}{f_{j}},$$

where $C_{i}$ represents the number of CPU cycles required to execute task *i* successfully.

In this paper, we assume orthogonal multiple access (OMA)-based device-to-device (D2D) communication between task owners and ENs. Task owners use orthogonal channels for input data transmission, and hence, the inter-user interference could be omitted [35]. With the interference-free OMA based networks, the achievable transmission data rate and the preference of each agent are, therefore, independent of other agents’ choices.

On the other hand, the transmission delay is given as follows:

(5) $$L_{ij}^{tra}=\frac{M_{i}}{B\log\left(1+\frac{p_{i}^{\mathrm{tra}}\gamma_{ij}\left(t\right)}{N_{0}}\right)},$$

where $M_{i}$ denotes the input data size of task *i*; $\gamma_{ij}\left(t\right)$ is the channel power gain from the owner of task *i* to EN *j* in the *t*-th time slot; $p_{i}^{\mathrm{tra}}$ is the transmission power; *B* is the system bandwidth; $N_{0}$ is the noise power spectral density at the receiver; and $B\log\left(1+\frac{p_{i}^{\mathrm{tra}}\gamma_{ij}\left(t\right)}{N_{0}}\right)$ represents the transmission throughput from the owner of task *i* to EN *j*. When the task is computed locally, the transmission delay is 0.

Intuitively, for computation-intensive tasks, the computation delay is responsible for a large portion of the overall delay. Hence, computation-intensive tasks tend to find an EN with higher computational capability to meet their latency constraints, while other kinds of tasks may prefer to be computed locally to save the extra transmission delay incurred by task offloading.

### 3.3. Energy Consumption

Then, we consider the energy consumption which is another major concern that needs to be addressed in task assignment. The total energy consumption for executing task *i* on EN *j* is denoted by $E_{ij}$ as follows:

(6) $$E_{ij}=E_{ij}^{\mathrm{tra}}+E_{ij}^{\mathrm{com}},$$

where $E_{ij}^{\mathrm{tra}}$ and $E_{ij}^{\mathrm{com}}$ represent the transmission energy consumption and computation energy consumption of EN *j*, respectively.

On one hand, the transmission energy consumption of the owner of task *i* is given as follows:

(7) $$E_{ij}^{\mathrm{tra}}=p_{i}^{\mathrm{tra}}L_{ij}^{\mathrm{tra}}=\frac{M_{i}p_{i}^{\mathrm{tra}}}{B\log\left(1+\frac{p_{i}^{\mathrm{tra}}\gamma_{ij}\left(t\right)}{N_{0}}\right)}.$$

The transmission energy consumption is 0 in case of the local computation mode.

On the other hand, the computation energy consumption of EN *j* is given as follows:

(8) $$\begin{array}{c} E_{ij}^{\mathrm{com}}=p_{j}^{\mathrm{com}}L_{ij}^{\mathrm{com}}=\frac{C_{i}p_{j}^{\mathrm{com}}}{f_{j}}, \end{array}$$

where $p_{j}^{\mathrm{com}}$ (in Joules per second) represents its energy consumption level.

### 3.4. Utility Function and Optimization Problem

In a matching game, a utility function is used to capture the net benefit that a task or an EN can receive from a task assignment. To reduce the overall energy consumption, we first define the utility of task *i* if it is computed on EN *j* as follows:

(9) $$\begin{array}{c} u_{ij}=r_{i}-\alpha E_{ij}^{\mathrm{tra}}-\lambda C_{i}, \end{array}$$

where $r_{i}$ is the owner’s reservation price (i.e., satisfaction) if task *i* can be finished within the designated delay tolerance; α is the energy cost coefficient; and γ is the unit price per CPU cycle that the owner of task *i* pays for the computation service offered by a remote EN. Note that the overall payment, $\lambda C_{i}$, is proportional to the size of the task, $C_{i}$.

Then, the utility of EN *j* achieved by completing task *i* is given as follows:

(10) $$\begin{array}{c} v_{ij}=\lambda C_{i}-\alpha E_{ij}^{\mathrm{com}}. \end{array}$$

Resource-limited devices seek to offload their computations to nearby ENs while taking into account the devices’ and MEC servers’ computational capabilities, wireless channel conditions, and delay constraints. The fundamental problem under consideration is cast as a network-wide utility maximization problem for task assignment, subject to delay requirements. Summing up the utility of all tasks and ENs over discreet $x_{ij}$, we have  

(11) $$\begin{array}{cc} & \sum_{i=1}^{N}\sum_{j=1}^{N}\left(u_{ij}+v_{ij}\right)x_{ij}\\ = & \sum_{i=1}^{N}\sum_{j=1}^{N}\left(r_{i}-\alpha E_{ij}^{\mathrm{tra}}-\alpha E_{ij}^{\mathrm{com}}\right)x_{ij}\\ = & \sum_{i=1}^{N}\sum_{j=1}^{N}r_{i}x_{ij}-\alpha \sum_{i=1}^{N}\sum_{j=1}^{N}\left(E_{ij}^{\mathrm{tra}}+E_{ij}^{\mathrm{com}}\right)x_{ij}. \end{array}$$

Hence, the problem of maximizing the overall utility is equivalent to minimizing the overall energy consumption:

(12) $$\begin{array}{c} \arg\max_{\left\{x_{ij}\right\}}\sum_{i=1}^{N}\sum_{j=1}^{N}\left(u_{ij}+v_{ij}\right)x_{ij}=\arg\min_{\left\{x_{ij}\right\}}\sum_{i=1}^{N}\sum_{j=1}^{N}\left(E_{ij}^{\mathrm{tra}}+E_{ij}^{\mathrm{com}}\right)x_{ij}. \end{array}$$

Our delay-constrained energy consumption optimization problem is defined as follows:

(13) $$\begin{array}{cc} & \min_{\left\{x_{ij}\right\}}\sum_{i=1}^{N}\sum_{j=1}^{N}\left(E_{ij}^{\mathrm{tra}}+E_{ij}^{\mathrm{com}}\right)x_{ij}\\ s.t.\; & (1)\;\sum_{i=1}^{N}x_{ij}\leq 1,\forall j\in \left\{1,\ldots ,N\right\}\\ & (2)\;L_{ij}\leq L_{i}^{\mathrm{tol}},\forall i\in \left\{1,\ldots ,N\right\}\\ & (3)\;u_{ij}x_{ij}\geq 0,\forall i\in \left\{1,\ldots ,N\right\}\\ & (4)\;v_{ij}x_{ij}\geq 0,\forall i\in \left\{1,\ldots ,N\right\}\\ & (5)\;\frac{p_{i}^{\mathrm{tra}}\gamma_{ij}\left(t\right)}{N_{0}}\geq x_{ij}\Gamma ,\forall i\in \left\{1,\ldots ,N\right\}\;\mathrm{and}\;i\neq j\\ & (6)\;x_{ij}\in \left\{0,1\right\},\forall i\in \left\{1,\ldots ,N\right\}\;\mathrm{and}\;\forall j\in \left\{1,\ldots ,N\right\}.\; \end{array}$$

Condition (1) guarantees that each task will be assigned to only one EN; Condition (2) guarantees that each task will be completed on time; Conditions (3) and (4) imply that the utility of each task and EN should not be negative; Condition (5) implies that the SNR should be higher than a threshold value to guarantee a successful transmission (reliable transmission constraint). The optimization problem shown in Equation (13) is in the standard form of the binary linear programming problem (BLP) with a bundle of constraints, which is one of Karp’s 21 problems and is proven to be NP-hard [36].

## 4. Matching-Theory Based Solution and Stability Analysis

Since the optimization problem in (13) is NP-hard, many problem instances are intractable, and so. heuristic methods should be used instead. In this section, we give our task assignment algorithm which is a highly practical model. The purpose of the proposed algorithm is to obtain a solution to the optimization problem in (13) in a distributive way.

The specific details of the algorithm are described in Algorithm 1, which consists of an initialization stage, followed by multiple iterations. In this section, a brief description of the algorithm during iteration *t* is given.

**Algorithm 1** The proposed algorithm.
1:
**Step 1: Initialization**
2:Set $x_{ij}=0,\forall i\in \left\{1,...,M\right\},\forall j\in \left\{1,...,N\right\}$3:Construct the list of all tasks that are not matched which is denoted by $\tau^{\mathrm{unmatch}}$.4:Each EN *j* broadcasts its CPU frequency, $f_{j}$, to its neighboring mobile devices.5:Each task, *i*, constructs its preference list according to the utility function shown in Equation (14).6:
**Step 2: Task Owners’ Proposal**
7:Each $\tau_{i}\in \tau^{\mathrm{unmatch}}$ makes a proposal to the neighboring node, $\epsilon_{j}$, that ranks the first in its preference list. At the same time, $\tau_{i}$ announces its input data size, $M_{i}$; number of required CPU cycles, $C_{i}$; and delay tolerance, $L_{i}^{\mathrm{tol}}$, which are necessary for ENs’ decision-making.8:
**if** $|\Phi \left(\epsilon_{j}\right)|\leq q_{j}$ **then**
9:    **if** $v_{ij}\geq 0$, $\frac{p_{i}^{\mathrm{tra}}\gamma_{ij}\left(t\right)}{N_{0}}\geq \Gamma$ and $L_{ij}\leq L_{i}^{\mathrm{tol}}$ **then**10:        Accept the proposal; add *i* to $\phi_{j}^{\tau }$; delete $\tau_{i}$ from $\tau^{\mathrm{unmatch}}$.11:    **else**12:        Reject the proposal.13:    **end if**14:
**else if** $|\Phi \left(\epsilon_{j}\right)|==q_{j}$ **then**
15:    **if** $v_{ij}\geq v_{i^{\prime }j}$, $\frac{p_{i}^{\mathrm{tra}}\gamma_{ij}\left(t\right)}{N_{0}}\geq \Gamma$ and $L_{ij}\leq L_{i}^{\mathrm{tol}},\forall i^{\prime }\in \phi_{j}^{\tau }$ **then**16:        Accept the proposal; add *i* to $\phi_{j}^{\tau }$; delete $\tau_{i}$ from $\tau^{\mathrm{unmatch}}$.17:    **else**18:        Reject the proposal.19:    **end if**20:
**end if**
21:**if**$\tau_{i}$ is not matched to any node **then**22:    There is no EN that can meet the delay requirements of $\tau_{i}$. Delete $\tau_{i}$ from $\tau^{\mathrm{unmatch}}$.23:
**end if**
24:Step 3: **End**25:**while**$\tau^{\mathrm{unmatch}}$ is not empty. **do**26:    Go to Step 2.27:
**end while**



### 4.1. Preference List and Proposal

Before the execution of matching, the first step is to setup the preference list for all tasks. The preference is evaluated based on local information which is defined as a utility function that qualifies the benefit achieved by certain task–EN matching shown in Equation (9). By substituting Equation (7) into Equation (9), we have

(14) $$\begin{array}{c} u_{ij}=r_{i}-\frac{\alpha M_{i}p_{i}^{\mathrm{tra}}}{B\log\left(1+\frac{p_{i}^{\mathrm{tra}}\gamma_{ij}\left(t\right)}{N_{0}}\right)}-\lambda C_{i}. \end{array}$$

We use the notation $\epsilon_{j}{≻}_{\tau_{i}}\epsilon_{l}$ to imply that task *i* is preferred to be executed on EN *j* over *l*, namely,

(15) $$\begin{array}{c} \epsilon_{j}{≻}_{\tau_{i}}\epsilon_{l}\Leftrightarrow u_{ij}\geq u_{il}. \end{array}$$

Let $\phi_{i}^{\epsilon }$ denote the set of indexes of ENs to which the input data of task *i* can be reliably transferred.

(16) $$\begin{array}{c} \phi_{i}^{\epsilon }=\left\{j^{\prime }|\frac{p_{i}^{\mathrm{tra}}\gamma_{ij^{\prime }}\left(t\right)}{N_{0}}\geq x_{ij^{\prime }}\Gamma \right\} \end{array}$$

Each task ranks the ENs in order of preference. Each task owner firstly proposes its most preferred EN in $\phi_{i}^{\epsilon }$ which results in the highest utility. Once this is done, the ENs that have received proposals reject all but their top suitor. Every task that is still not matched proposes to his most preferred EN which has not already rejected it.

### 4.2. Accept/Reject Proposal

On receiving a proposal from the owner of task *i*, the EN also has to make its decision on whether to accept or reject the proposal in order to maximize its own utility. Each EN can accept, at most, its quota of tasks. The philosophy of each EN’s decision-making is described case by case as follows:

- **Case 1**: The number of tasks which has been matched to EN *j* is less than or equal to its quota, i.e., $|\Phi \left(\epsilon_{j}\right)|\leq q_{j}$.
  Since the EN has idle resources, it simply accepts $\tau_{i}$, which gives it non-negative utility; otherwise, it rejects $\tau_{i}$.
- **Case 2**: The number of tasks which has been matched to EN *j* is equal to its quota, i.e., $|\Phi \left(\epsilon_{j}\right)|=q_{j}$.
  Since there is no quota left, it accepts $\tau_{i}$ if it prefers to include $\tau_{i}$ over its current matches; otherwise it rejects $\tau_{i}$.

We continue until all task are matched or the delay requirements of the unmatched tasks cannot be satisfied by any ENs. The runtime complexity of this algorithm is $O(N^{2})$, where *N* is the number of tasks or ENs.

### 4.3. Stability Analysis

Given the task set, τ, and EN set, ϵ, the key question is whether we can pair up the tasks and ENs in a stable fashion? In task assignment for MEC systems, the matching stability gives robustness to deviations that can benefit both the task owners and the ENs. Formally, in stable matching, there are no task-EN pairs that both prefer each other over their current match.

**Lemma** **1.** *When the proposed algorithm terminates, we have a stable matching between the tasks and ENs.*

**Proof.**  Let $\tau_{i}$ and $\epsilon_{j}$ both be matched, but not to each other. Upon completion of the algorithm, it is not possible for both $\tau_{i}$ and $\epsilon_{j}$ to prefer each other over their current partners. If $\tau_{i}$ prefers $\epsilon_{j}$ to its current partner, $\tau_{i}$ must have proposed to $\epsilon_{j}$ before it proposed to its current partner. If $\epsilon_{j}$ accepts its proposal, yet is not matched to $\tau_{i}$ at the end, $\epsilon_{j}$ must have dumped $\tau_{i}$ for another task that it prefers more, and therefore, it does not prefer $\tau_{i}$ more than its current partner. If $\epsilon_{j}$ rejects $\tau_{i}$’s proposal, $\epsilon_{j}$ was already with a task that it prefers more than $\tau_{i}$. ☐

### 4.4. Practical Implementation

The matching decision process is intrinsically distributed (i.e., no centralized coordination is needed), in that each agent selfishly maximizes its utility based on its locally collected information. From the task owners’ perspective, in order to construct the preference list, they require knowledge of the computation capability of the neighboring ENs. From the ENs’ perspective, in order to construct the preference list and judge whether the computation capability is enough to satisfy the delay constraint of the task, they need to know the information about the input data size, the number of required CPU cycles, and the delay tolerance of the task. All of this information is exchanged between the ENs and task owners at the initialization stage.

## 5. Numerical Evaluation

Simulations were conducted to evaluate the performance of the proposed scheme. We simulated a 50 m × 50 m square area with 10 ENs randomly deployed in the area. The input data size and the number of required CPU cycles of each task were randomly selected within the range of [400, 800] kB, and [1, 5] $\times 10^{9}$, respectively. The delay tolerance of each task was set to 1.5 s. The transmission power ($p_{i}^{\mathrm{tra}}$) and computing energy consumption level ($p_{i}^{\mathrm{com}}$), were set to 36 dBm and 40 W, respectively. Moreover, the CPU frequency of each EN was randomly selected within the range of [2, 4] GHz. Other simulation settings can be found in Table 1. We used a random matching scheme, a computational capability based matching (CCBM) scheme, and an exhaustive matching scheme for comparison.

- Proposed scheme: Tasks are intelligently matched to ENs by taking into account both the energy consumption and the delay constraints.
- Random matching scheme: Tasks and ENs are randomly paired together as long as all conditions in Equation (13) are satisfied.
- CCBM scheme: The EN with higher computational capability has a higher priority to accept tasks as long as it can meet all conditions shown in Equation (13).
- Exhaustive matching scheme: A centralized authority with complete information searches through all possible combinations to find the optimum solution.

**Figure 3:**
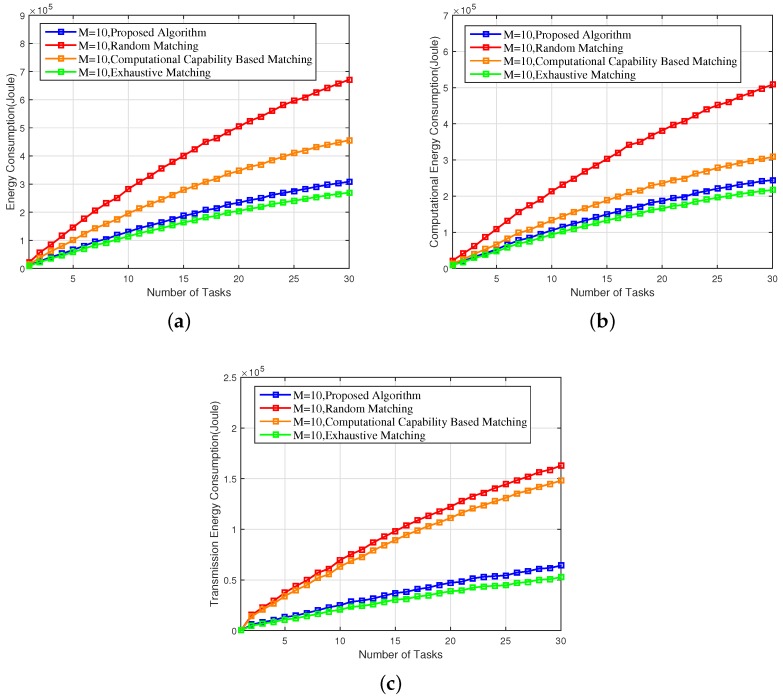
(**a**) Overall energy consumption, (**b**) computational energy consumption, and (**c**) transmission energy consumption of the proposed scheme versus the random matching, CCBM, and exhaustive matching.

Figure 3a–c depict the overall energy consumption, the computational energy consumption, and the transmission energy consumption with a varying number of tasks. Compared to the random matching scheme and the CCBM scheme, the overall energy consumption of the proposed scheme reduced by 53.69% and 32.78%; the computational energy consumption of the proposed scheme reduced by 50.97% and 20.62%; and the transmission energy consumption of the proposed scheme reduced by 62.15% and 58.39%, respectively. This is not surprising, because in our proposed scheme, each agent (task or EN) ranks the agents in the opposite set based on a utility function that captures the energy consumption (computational energy consumption or transmission energy consumption), and our matching algorithm in nature guarantees the energy efficiency. Although the computational energy consumption is also taken into account when pairing up the ENs and tasks in the CCBM scheme, ENs with higher computational energy efficiency are likely to starve ENs with a lower computational energy efficiency but higher transmission energy efficiency, which is evidenced by the large gap between the proposed scheme and the CCBM scheme in terms of transmission energy consumption.

**Figure 4:**
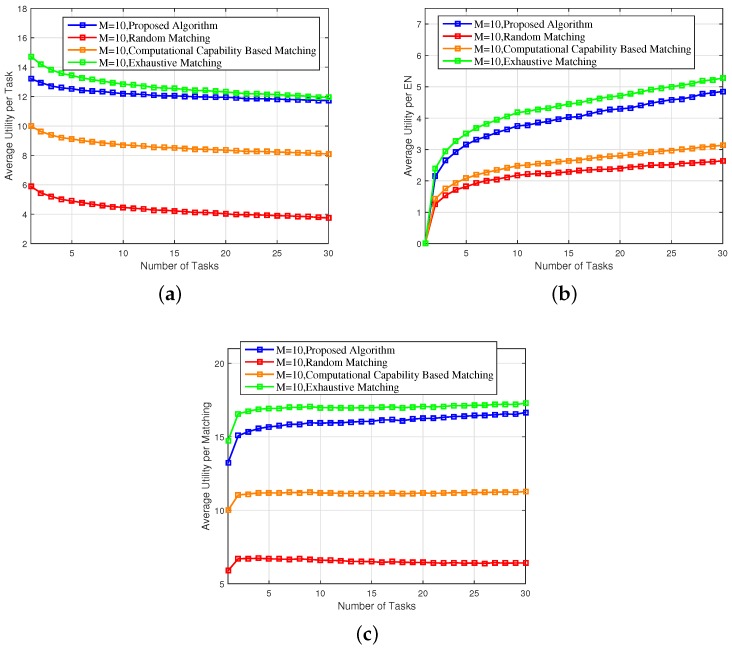
(**a**) Average utility per task, (**b**) average utility per EN, and (**c**) average utility per matching of the proposed scheme versus the random matching, CCBM, and exhaustive matching

Figure 4a–c depict the average utility per task, the average utility per EN, and the average utility per matching with varying number of tasks. As we can observe from Figure 3, the average utility per matching increases with the number of tasks and gradually converges to a maximum value. When the number of tasks is relatively small, tasks can easily find energy-efficient ENs to pair with. Hence, the average utility per matching increases with the number of tasks. When the number of tasks is large, the capacity of ENs becomes insufficient to accommodate all tasks. Hence the average utility per matching gradually converges to a maximum value. Compared to the random matching scheme and the CCBM scheme, the average utility per task of the proposed scheme increased by 179.57% and 40.79%; the average utility per EN of the proposed scheme increased by 77.16% and 52.72%; and the average utility per matching of the proposed scheme increased by 128.37% and 46.76%, respectively. The performance gain is also achieved through intelligently offloading tasks to the ENs with better wireless channel conditions and computation efficiency.

**Figure 5:**
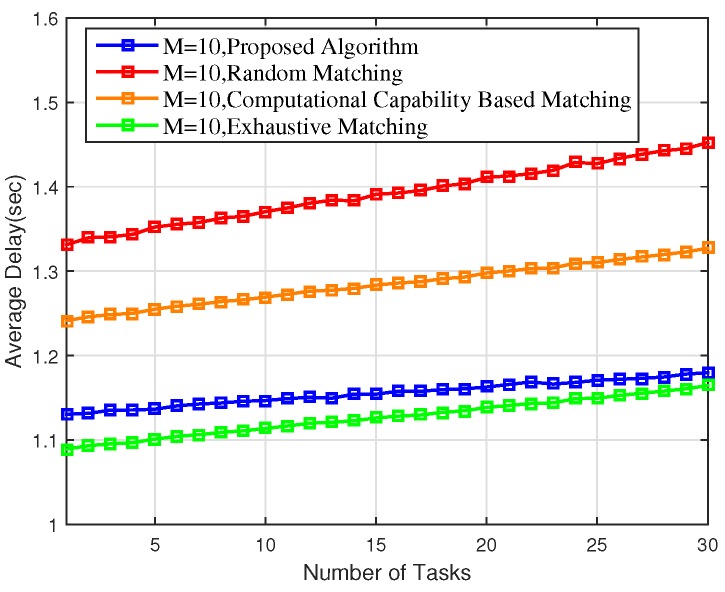
Average delay of the proposed scheme versus the random matching, CCBM, and exhaustive matching.

In practice, different tasks have different time sensitivities. For computation-intensive tasks, computation delay is responsible for a large portion of the overall delay. Hence, computation-intensive tasks tend to find an EN with higher computational capability to meet their latency constraints, while other kinds of tasks may prefer to be computed locally to save the extra transmission delay incurred by task offloading. Intelligent task offloading can therefore largely reduce the latency. As shown in Figure 5, the average delay per task of the proposed scheme reduced by 17% and 10.05% compared to the random matching scheme and the CCBM scheme, respectively.

**Figure 6:**
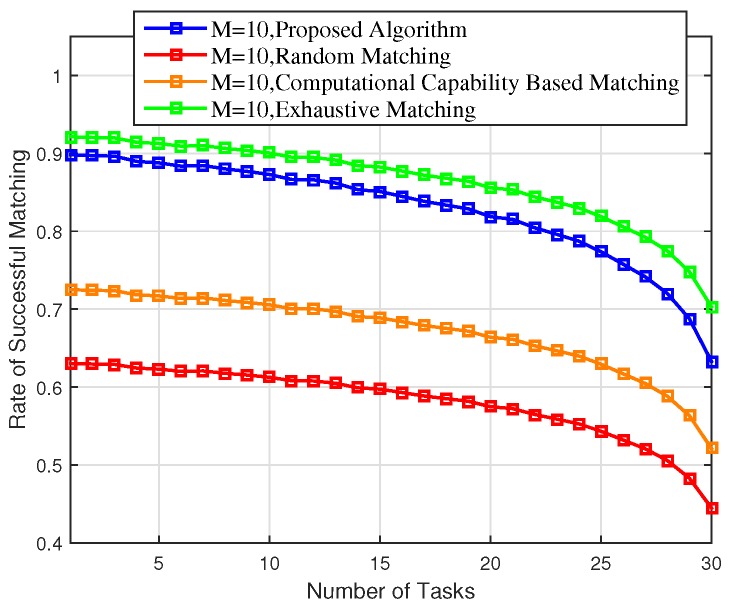
Rate of successful matching of the proposed scheme versus the random matching, CCBM, and exhaustive matching.

Figure 6 shows that the rate of successful matching decreased with the number of tasks. There are various reasons that can contribute to an unsuccessful matching, for example, there is no capacity left to accommodate the computation task or there is no EN that can meet the task’s delay requirement. In our simulation, we assumed that there were ten ENs and each of them was able to accept two tasks on average. It was observed that the rate of successful matching decreased dramatically when the number of tasks was larger than the total capacity (i.e., 20 = 10 ENs × 2 tasks per EN).

**Figure 7:**
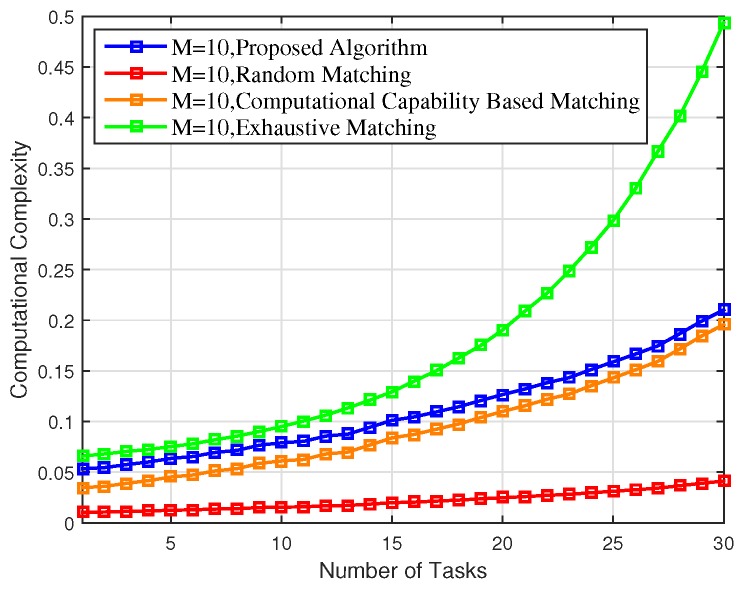
Computational complexity of the proposed scheme versus the random matching, CCBM, and exhaustive matching.

Nevertheless, all the performance gains of the proposed scheme come at the cost of interactive decision-making overhead and consequent computational complexity, as shown in Figure 7.

Finally, as can be observed from Figure 3, Figure 4, Figure 5, Figure 6 and Figure 7, the proposed scheme offers a comparable performance to the exhaustive scheme, with only 14.36%, 5.43%, and 3.37% differences in terms of overall energy consumption, average utility per matching, and average delay, respectively, while the proposed scheme significantly outperforms the exhaustive matching scheme in terms of reducing the computational complexity by 64.01%.

## 6. Conclusions and Future Work

In this paper, we presented the first matching theory based task assignment mechanism for MEC. We formulated the task assignment problem with the objective of minimizing the energy consumption by taking into account the devices’ and MEC servers’ computational capabilities, wireless channel conditions, and delay constraints, and proposed a heuristic-based algorithm that easily includes heuristics in the problem to solve this minimization problem. Simulations were carried out to evaluate the efficiency of the proposed mechanism. The results showed that our proposed scheme provides a good compromise between computational complexity and energy consumption optimization.

In this work, we assumed that ENs use orthogonal channels for input data transmission, and hence, the inter-user interference was omitted. However, in non-orthogonal multiple access networks, which are regarded as a radio access candidate for the fifth-generation (5G) wireless, when more ENs in the proximity use the same spectrum for input data transmission, the inter-user interference level increases, and hence, the achievable data rate decreases. As a key consequence, the performance and the preference order of each agent is strongly affected by the dynamic formation of other task-EN associations which is called externality. If such externality is not well managed, an agent may have to keep changing its preference order responsive to the changes in other task-EN associations, and a stable result can never be expected. The externality issue will be addressed in our future studies.

## Figures and Tables

**Table 1:** Simulation settings.

Simulation Parameter	Value
Number of ENs (*N*)	10
Input data size ($M_{i}$)	[400, 800] kB
Number of required CPU cycles ($C_{i}$)	[1, 5] $\times \;10^{9}$
Delay tolerance ($L_{i}^{\mathrm{tol}}$)	1.5 s
Maximum transmission power ($p_{i}^{\mathrm{tra}}$)	36 dBm
Energy consumption level ($p_{j}^{\mathrm{com}}$)	[2, 20] W
Quota ($q_{j}$)	1, 2, or 3
System bandwidth (*B*)	20 MHz
Channel power gain ($\gamma_{ij}$)	$-40\;d^{-4}$ (dB)
CPU frequency ($f_{j}$)	[2, 4] GHz
Noise power spectral density ($N_{0}$)	−110 dBm
Energy cost coefficient (α)	$10^{-3}$
Unit price per CPU cycle (λ)	$2\times 10^{-9}$
SINR threshold value (Γ)	20 dB

## Abbreviations

The following abbreviations are used in this manuscript:

AR	Augmented Reality
MEC	Mobile Edge Computing
EN	Edge Node
VRU	Virtual Resource Unit
QoS	Quality of Service
QoE	Quality of Experience
BLP	Binary Linear Programming
RACS	Radio Applications Cloud Server
SDN	Software Defined Networking
VANETs	Vehicular Ad Hoc Networks
D2D	Device-to-Device
